# Type I Cryoglobulinemia Associated With Multiple Myeloma: A Case Report

**DOI:** 10.7759/cureus.90589

**Published:** 2025-08-20

**Authors:** Odalyvianey Reyes-Báez, Ana L Carrillo-González, Salma Triana-González, Abihai Lucas-Hernandez, Olga Vera Lastra

**Affiliations:** 1 Internal Medicine, Hospital de Especialidades "Dr. Antonio Fraga Mouret", Centro Médico Nacional "La Raza", Instituto Mexicano del Seguro Social, Mexico City, MEX; 2 Infectious Disease, Hospital de Especialidades "Dr. Antonio Fraga Mouret", Centro Médico Nacional "La Raza", Instituto Mexicano del Seguro Social, Mexico City, MEX; 3 Rheumatology, Hospital General de Teziutlán, Teziutlán, MEX

**Keywords:** cryoglobulins, hyperviscosity syndrome, multiple myeloma, pseudovasculitis, type i monoclonal cryoglobulinemia

## Abstract

Cryoglobulinemia is a rare disorder characterized by the presence of serum cryoglobulins, which may result from clonal B-cell expansion (Type I), chronic infections, or autoimmune diseases (mixed cryoglobulinemia: Types II and III). We report the case of an 83-year-old man who fulfilled the diagnostic criteria for Type I cryoglobulinemic vasculitis, presenting with confluent purpuric macules and papules, some ulcerated, along with necrosis. Skin biopsy revealed thrombotic vasculopathy, and laboratory evaluation identified an IgG kappa light-chain monoclonal gammopathy, confirming multiple myeloma as the underlying etiology. Given its rarity and potential for severe complications, early and accurate diagnosis is crucial. In this case, targeted therapy for the underlying plasma cell disorder led to marked improvement of cutaneous lesions and resolution of hyperviscosity symptoms within two months.

## Introduction

Cryoglobulins are plasma proteins that precipitate at temperatures below 37 °C. They develop either from clonal B-cell proliferation, chronic infection, or autoimmune-driven immune stimulation. The term “cryoglobulinemic vasculitis” refers to clinical signs directly related to cryoglobulin deposits in blood vessels. According to Brouet’s classification, cryoglobulinemia is categorized into three types: Type I (10-15%), which involves monoclonal immunoglobulins without rheumatoid-factor activity; Type II (65%), involving monoclonal IgM with rheumatoid-factor activity plus polyclonal IgG; and Type III (25%), characterized by polyclonal IgM and IgG with rheumatoid-factor activity [1,2].

Type I cryoglobulinemia is most often linked to B-cell proliferative disorders, particularly Waldenström macroglobulinemia, chronic lymphocytic leukemia, and multiple myeloma (MM), which is the second most common hematologic malignancy [3]. Overproduction of monoclonal immunoglobulins (IgG, IgM, IgA) and/or free light chains (kappa or lambda) can be identified by serum immunofixation or urine protein electrophoresis [4]. Without timely recognition and treatment, Type I cryoglobulinemia may result in irreversible organ damage and significant mortality, underscoring the importance of early diagnosis.

The primary skin manifestations of cryoglobulinemia include ulcers and necrosis in 5-25% of cases, and it has been associated with MM in up to 10% [5]. Despite its rarity, prompt recognition and treatment are crucial, as delayed diagnosis can worsen organ damage. Here, we present a clinical case and review the relevant literature.

## Case presentation

**Figure 1:**
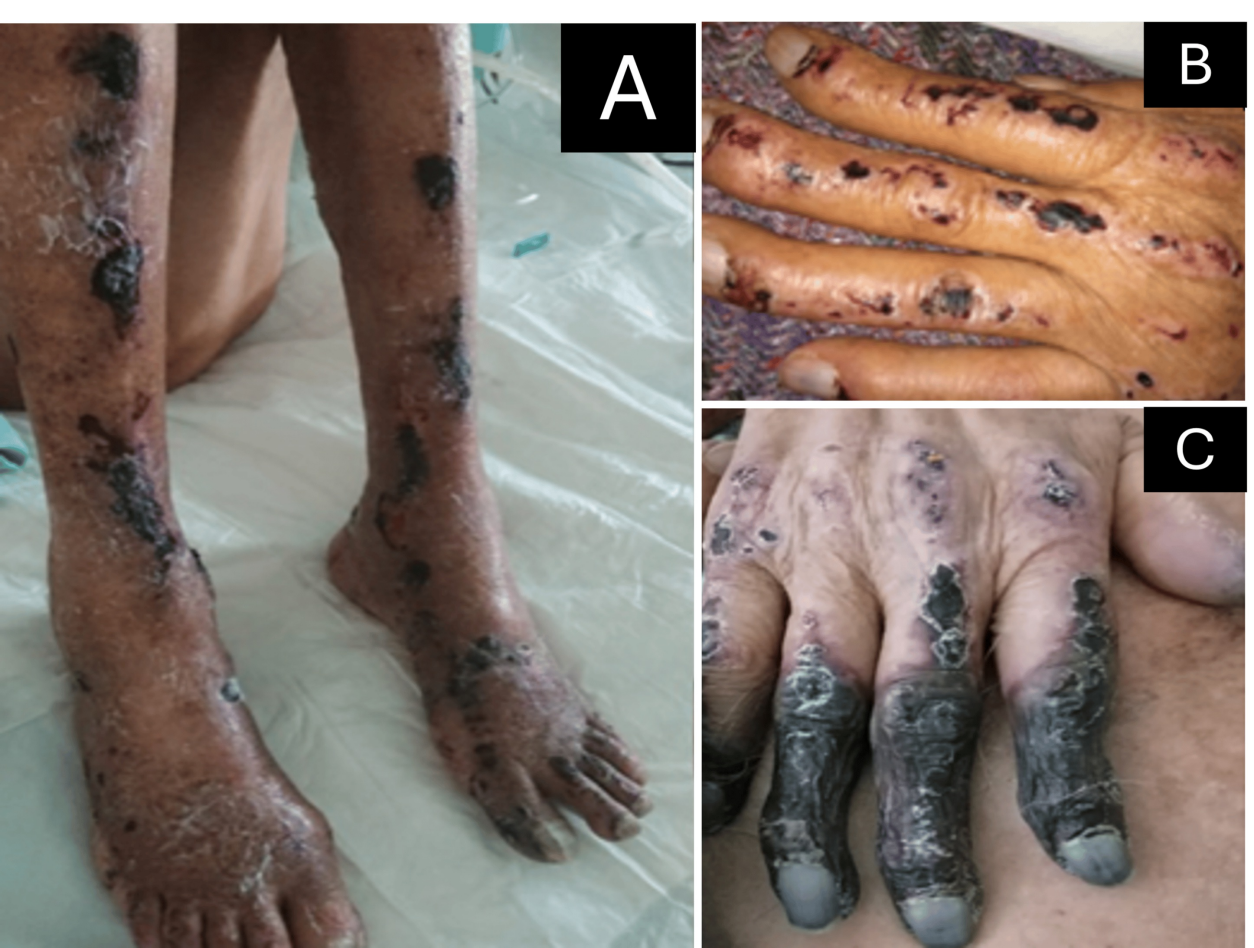
(A) Violaceous macules on the lower extremities with crusts, without signs of arterial compromise. (B) Violaceous macules on the back of the left hand. (C) Necrosis affecting the second through fifth fingers and dorsum of the right hand.

An 83-year-old man presented to the continuous admission unit of our hospital and was subsequently admitted to the internal medicine ward. His chief complaint was the appearance of painful purpuric skin lesions on the scalp, upper, and lower extremities, which had begun approximately three weeks earlier and had progressively worsened. The lesions were associated with exposure to cold and evolved from macules to papules, some of which ulcerated, and were associated with acral necrosis affecting the second through fifth digits of the right hand. He also reported progressive weakness, intermittent headaches, and dyspnea over the preceding week. There were no prior episodes of similar lesions. The patient denied previous hospitalizations for this condition and had not received specific treatment before presentation. He had a history of bone pain and recent hypercalcemia but no known autoimmune diseases. His past medical history was otherwise unremarkable, and he was not taking any chronic medications at the time of admission. On physical examination, lesions were characterized by non-blanching purpuric macules and papules measuring approximately 5 mm (0.5 cm) in diameter, confluent in some areas, with occasional ulceration and necrosis of the digits (Figure 1). The distribution of the lesions in acral areas, their worsening with cold exposure, and the presence of necrosis raised the suspicion of cryoglobulinemic vasculopathy; therefore, serum cryoglobulin testing was ordered. During hospitalization, he developed a severe headache, reduced alertness (Glasgow Coma Scale score: 11/15), and dyspnea. A brain computed tomography (CT) scan was unremarkable. Serum cryoglobulins were detected, and serum viscosity was elevated; therefore, hyperviscosity syndrome was diagnosed. The rest of the laboratory tests are shown in Table 1. He underwent three sessions of plasmapheresis, resulting in symptomatic remission.

**Table 1:** Laboratory test results ANA: Antinuclear antibodies; anti-dsDNA: anti-double-stranded DNA; anti-CCP: anti–cyclic citrullinated peptide; RF: rheumatoid factor; HIV: human immunodeficiency virus; HBV: hepatitis B virus; HCV: hepatitis C virus

Parameter	Result	Reference Range
Hemoglobin	11.0 g/dL	12–16 g/dL (men)
Leukocytes	106.5 × 10³/µL	4.0–11.0 × 10³/µL
Platelets	250 × 10⁹/L	150–400 × 10⁹/L
Total Protein	10.0 g/dL	6.0–8.3 g/dL
Albumin	2.9 g/dL	3.5–5.0 g/dL
Serum Creatinine	1.36 mg/dL	0.6–1.3 mg/dL
Calcium	10.8 mg/dL	8.5–10.5 mg/dL
β2-microglobulin	6 mg/dL	≤2.5 mg/L
Bone Marrow Plasma Cells	57%	<5%
HIV, HBV, HCV Serologies	Negative	Negative
ANA, anti-dsDNA, anti-Ro/La, anti-CCP, RF	Negative	Negative

**Figure 2:**
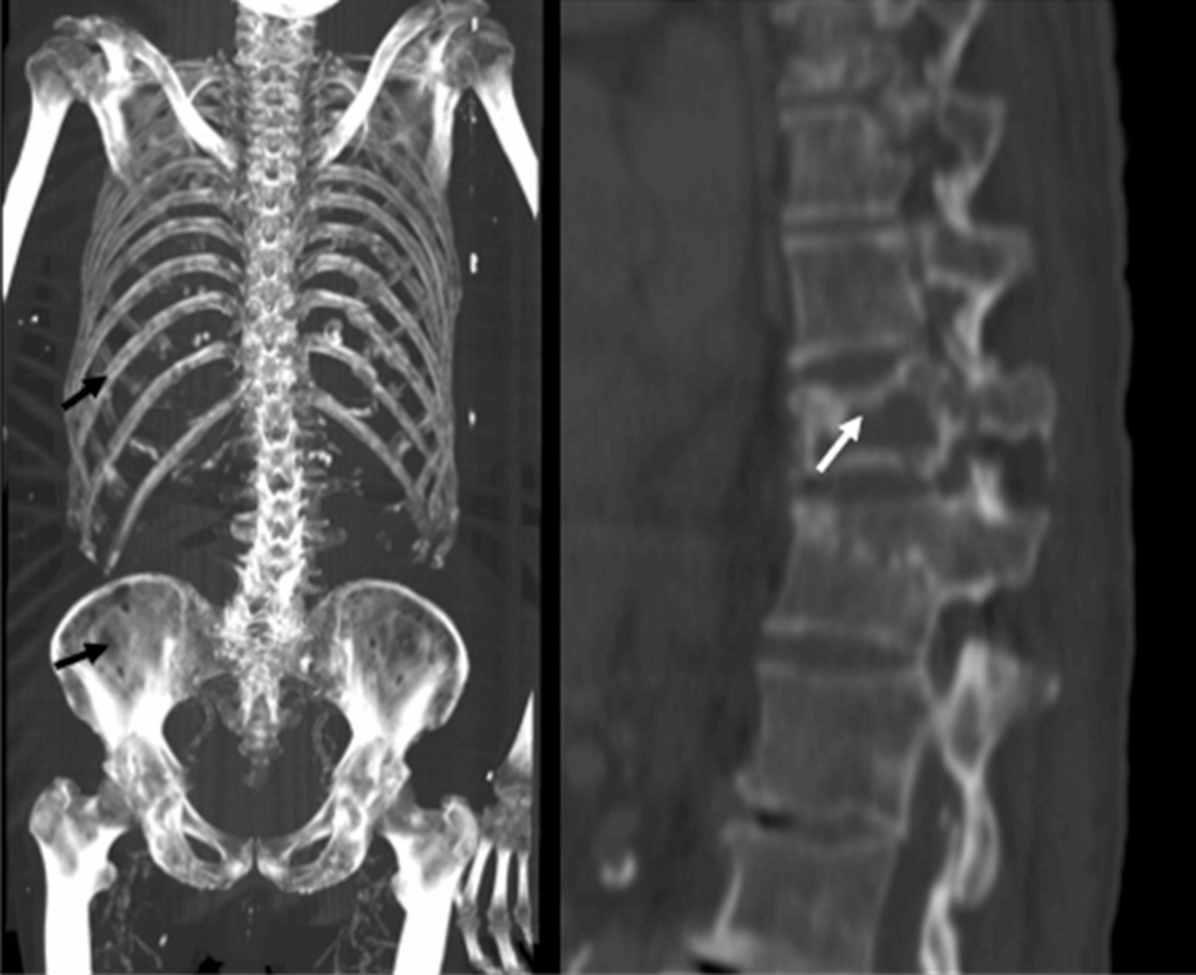
Computed tomography scan showing multiple osteolytic lesions in the ribs, vertebral bodies, and pelvis (arrows).

**Figure 3:**
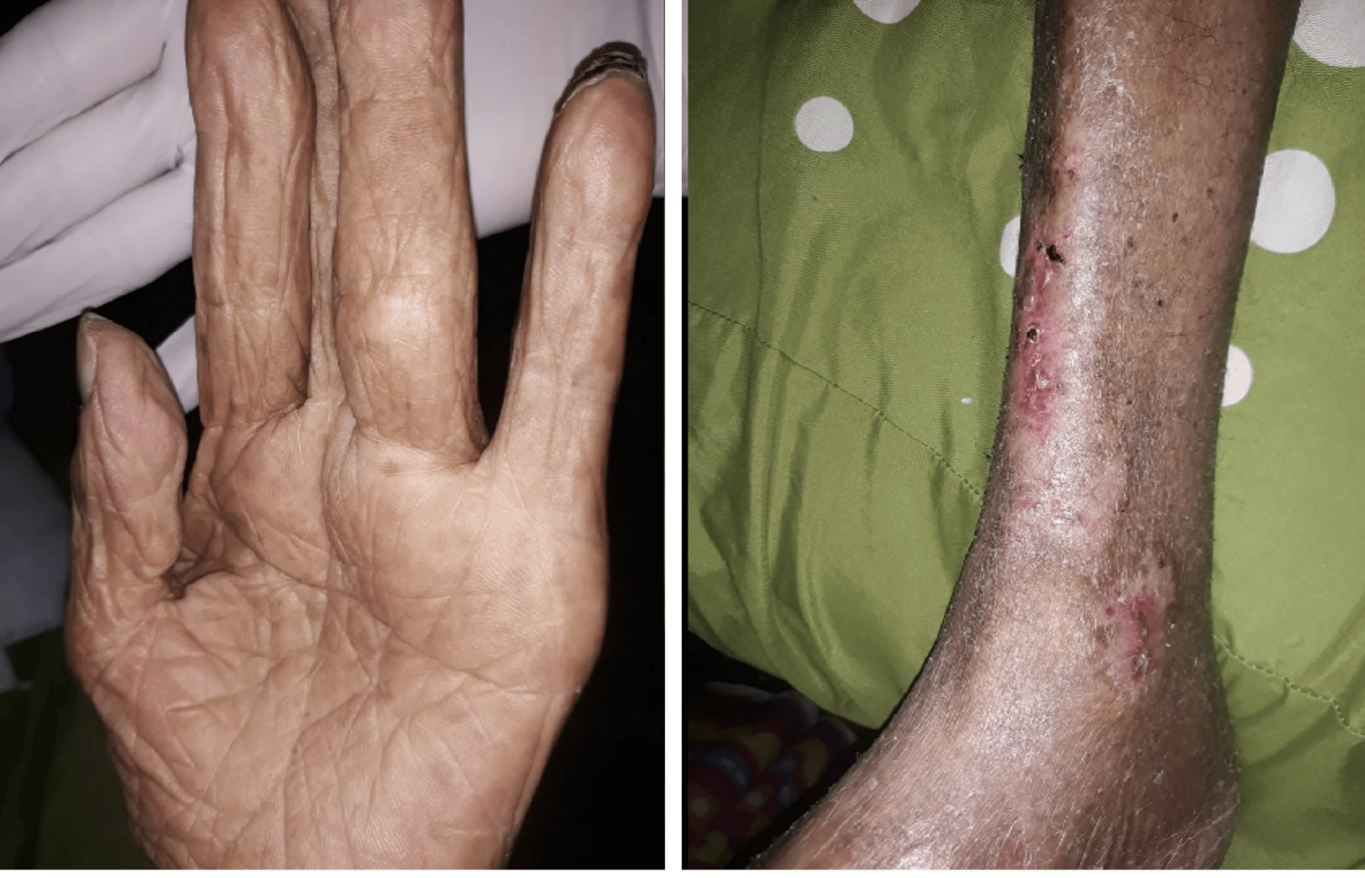
Remission of lesions on the left hand and lower extremities following treatment.

Given his history of bone pain and hypercalcemia, a CT scan was performed, revealing diffuse lytic lesions in the ribs, vertebrae, and pelvis (Figure 2). Serum protein electrophoresis on cellulose acetate membranes showed a gamma-region monoclonal spike of 0.9 g/dL (19%), and immunofixation confirmed an IgG kappa monoclonal gammopathy. Punch biopsy of a skin lesion demonstrated thrombotic vasculopathy with dense neutrophilic infiltrate and fibrinoid necrosis, without true vasculitis. These findings supported a diagnosis of Type I cryoglobulinemia secondary to light-chain MM. Therapy was initiated with rituximab 375 mg/m² IV weekly for four weeks, plus monthly IV cyclophosphamide 750 mg. He was transitioned to outpatient oral melphalan, thalidomide, and a tapering prednisone regimen (starting at 50 mg daily). Despite his advanced age and male sex, both poor prognostic factors, targeting the underlying plasma cell disorder, led to marked improvement of cutaneous lesions within 6-8 weeks (Figure 3).

## Discussion

This patient exhibited Meltzer’s triad, characterized by arthralgias, weakness, and purpura, which occurs in up to 80% of cryoglobulinemia cases, reflecting acral ischemia caused by cold-induced vascular occlusion [1-6]. Type I cryoglobulinemia has an estimated prevalence of approximately 1 in 100,000, with a female-to-male ratio of 3:1 and a peak incidence between ages 45 and 65 [7]. In a series of 102 Type I patients, cutaneous manifestations were observed in 63% (purpura 42%, ulcers 34%, gangrene in severe cases); neurologic symptoms in 32% (predominantly sensory neuropathy); arthralgias in 24%; and glomerulonephritis in only 14%. Other skin findings include Raynaud phenomenon (25%), acrocyanosis, and livedo reticularis [2-8].

Diagnosis depends on detecting serum cryoglobulins [2]. Type I cryoglobulins (> 5 g/L) precipitate within hours and do not have rheumatoid-factor activity or complement activation [9]. Uncommonly, mixed cryoglobulinemias exhibit low C4 levels with normal C3; our patient displayed this pattern despite a Type I etiology. Differential diagnoses include hepatitis B/C-related mixed cryoglobulinemia and autoimmune vasculitides (ANCA‑associated, Sjögren syndrome, systemic lupus erythematosus), requiring viral panels and autoantibody testing [8]. Histopathology typically shows occlusive, hyaline thrombi in small vessels [1,2]. Hyperviscosity syndrome (causing neurologic, ocular, and ENT symptoms) needs urgent plasmapheresis [2].

Current guidelines recommend high-dose corticosteroids combined with cytotoxic agents as initial therapy in severe cases, with the addition of rituximab or novel agents depending on the underlying hematologic disorder [6]. Rituximab, thalidomide, and lenalidomide each demonstrate an effectiveness of 80-86% in resolving skin lesions and removing cryoglobulins, based on series including predominantly Type I cases but also mixed cryoglobulinemia [6-10]. In our patient, rituximab and cyclophosphamide were initiated promptly, followed by oral melphalan, thalidomide, and a tapering corticosteroid regimen, achieving complete resolution of hyperviscosity symptoms and significant cutaneous healing within 6-8 weeks. He remains under outpatient hematology follow-up with sustained clinical improvement.

The CryoVas study followed 64 patients, including 28 with monoclonal gammopathy, over a period of 1 to 10 years, and found survival rates of 97% at one year and 87% at 10 years [7]. Type I cryoglobulinemia secondary to monoclonal gammopathy has a better prognosis [1-4]. Nevertheless, it is influenced by several factors, with advanced age and male sex associated with worse outcomes [3,7]. In our patient, both factors were present; however, timely initiation of therapy directed at the underlying plasma cell disorder led to a rapid and marked improvement of cutaneous lesions within 6-8 weeks.

Type I cryoglobulinemia can lead to severe complications if not promptly diagnosed and treated. Reported manifestations include acral necrosis, chronic non-healing ulcers, peripheral neuropathy, glomerulonephritis, and, in cases with high cryoglobulin burden, hyperviscosity syndrome causing neurological and ocular symptoms [2,6,8]. Involvement of vital organs such as the kidneys or central nervous system is associated with increased morbidity and mortality. Prognosis largely depends on controlling the underlying clonal disorder; patients with Type I cryoglobulinemia secondary to MM generally have a poorer long-term outcome compared to those with monoclonal gammopathy of undetermined significance, particularly when advanced age, male sex, or significant tumor burden are present [3,7]. Early intervention targeting the plasma cell clone can significantly improve survival and reduce irreversible organ damage [6,10].

## Conclusions

Type I cryoglobulinemia is a rare cause of small-vessel occlusion caused by monoclonal immunoglobulin precipitation. In this case, an IgG kappa light-chain MM underlay thrombotic vasculopathy and hyperviscosity symptoms. Awareness of this rare presentation is essential, as early diagnosis and treatment of the underlying plasma cell disorder can prevent irreversible organ damage and significantly improve patient outcomes.
